# Current diagnosis and treatment of acute pancreatitis in China: a real-world, multicenter study

**DOI:** 10.1186/s12876-021-01799-1

**Published:** 2021-05-08

**Authors:** Chuandong Sun, Zhu Li, Zheng Shi, Guichen Li

**Affiliations:** 1 Department of Surgery, The Affiliated Hospital of Qingdao University, Shandong, People’s Republic of China; 2 Department of Surgery, The Affiliated Hospital of Guizhou Medical University, Guizhou, People’s Republic of China; 3 Department of Surgery, The First Affiliated Hospital of Fujian Medical University, Fujian, People’s Republic of China; 4 Department of Surgery, The First Hospital of China Medical University, No. 155 Najing North Street, Heping District, Shenyang, Liaoning Province People’s Republic of China

**Keywords:** Acute pancreatitis, Real-world, Somatostatin, Octreotide, Real-world evidence, Electronic medical records

## Abstract

**Background:**

Efficacy of pancreatic enzyme inhibitors in acute pancreatitis (AP) is unclear in China.

**Aims:**

We aimed to present the current status of AP and evaluate the efficacy of pancreatic enzyme inhibitors in a larger population in China.

**Method:**

A retrospective, cross-sectional, real-world, multicenter analysis of a large dataset of patients presenting with AP from four hospitals of China over a two-year period was performed. Data were collected from the existing clinical records and the patients were grouped into medication group (somatostatin or octreotide or somatostatin and octreotide) and no medication group. Pair wise propensity score matching was performed for comparing somatostatin, octreotide and somatostatin/octreotide. The end points were incidence of disease complications, organ failure, hospitalization duration, and recovery time taken (hours) for serum amylase/serum lipase to normalcy.

**Results:**

A total of 3900 patients were recruited and 2775 patients were included for analysis. A total of 1100, 661, 676 and 338 patients received either somatostatin or octreotide or somatostatin and octreotide or no medication, respectively. The incidence of complications (7.6% vs 13.6%), organ failure (4.5% vs 7.4%), and the instances of entering ICU (9.3% vs 13.3%) were higher in unmedicated group. Complications at discharge (2.91 times), organ failure (2.53 times), and hospitalization stay were higher in octreotide-treated patients compared with somatostatin-treated patients. In comparison to the octreotide group, the serum amylase/lipase recovery time was shorter in the somatostatin group.

**Conclusion:**

This real-world study suggested that the use of pancreatic enzyme inhibitors was positively associated with greater clinical efficacy in AP patients and somatostatin might be more effective than octreotide in real-world settings in China.

**Supplementary Information:**

The online version contains supplementary material available at 10.1186/s12876-021-01799-1.

## Introduction

Acute pancreatitis (AP) is a gastrointestinal disease caused by sudden edema of pancreas that may lead to multiorgan failure or death. In the past few decades, the incidence of AP is on the rise throughout the world [1–4]. In majority of patients (80%–85%), AP develops as a milder disease course, with recovery in 1–2 weeks and a mortality rate of < 1–3%. However, around 20–30% of AP patients develop severe acute pancreatitis (SAP) leading to a mortality rate of 13–35% [5, 6]. Further, SAP also leads to sepsis and multiple organ failure, which requires intensive care unit (ICU) admission and has a higher risk of death. Due to the advancement of intensive care and surgical procedures, mortality rate, length of hospitalization stay, and cost of hospitalization had decreased from 2011 to 2016, despite an increase in the number of hospitalized AP patients [7].

Generally, mild AP is treated using supportive care measures. Moderate AP and SAP are treated with pharmacological interventions to decrease the morbidity and mortality rates [8]. Somatostatin and its analogs were used as antisecretory agents and anti-inflammatory peptides that inhibit the digestive enzymes secreted in the pancreas [9]. Somatostatin or octreotide was used as monotherapy to treat moderate AP and SAP [10], both were reported to improve the mortality rate and complications of SAP [11]. The usage of pharmacological interventions, including somatostatin analogues, is not recommended in various national guidelines from Japan [12], Canada [13], and America [14] mainly due to the lack of quality clinical evidence. However, somatostatin and its analogues are recommended by various Chinese guidelines and consensus statement [15]. While multiple studies have evaluated the efficacy outcomes with multiple endpoints, the evidence base is still inconclusive and has limitations. The inconsistent recommendations for somatostatin analogs in clinical practice recommendations and guidelines for AP may be due to the lack of high-quality clinical evidence. Therefore, herein, we reviewed the medical records of AP patients from four different hospitals in the major regions of China and discussed the current status of AP patients and the therapeutic efficacy of somatostatin analogues.

## Methods

### Study design

This study was designed as a real-world, retrospective, cross-sectional, multicenter study on patients hospitalized with AP in different regions of China. Electronic medical records (EMR) from multiple hospitals in different regions of China (north, south, east, and west China) were extracted. The four hospitals were: The First Hospital of China Medical University, The First Affiliated Hospital of Fujian Medical University, The Affiliated Hospital of Qingdao Medical University, and The Affiliated Hospital of Guizhou Medical University. All these hospitals are tier-3 affiliated hospitals (hospitals of excellence) of medical universities. This study was approved by the respective ethical committee from the individual hospitals and was done in compliance with the Chinese laws before study initiation.

### Patient population

**Fig. 1:**
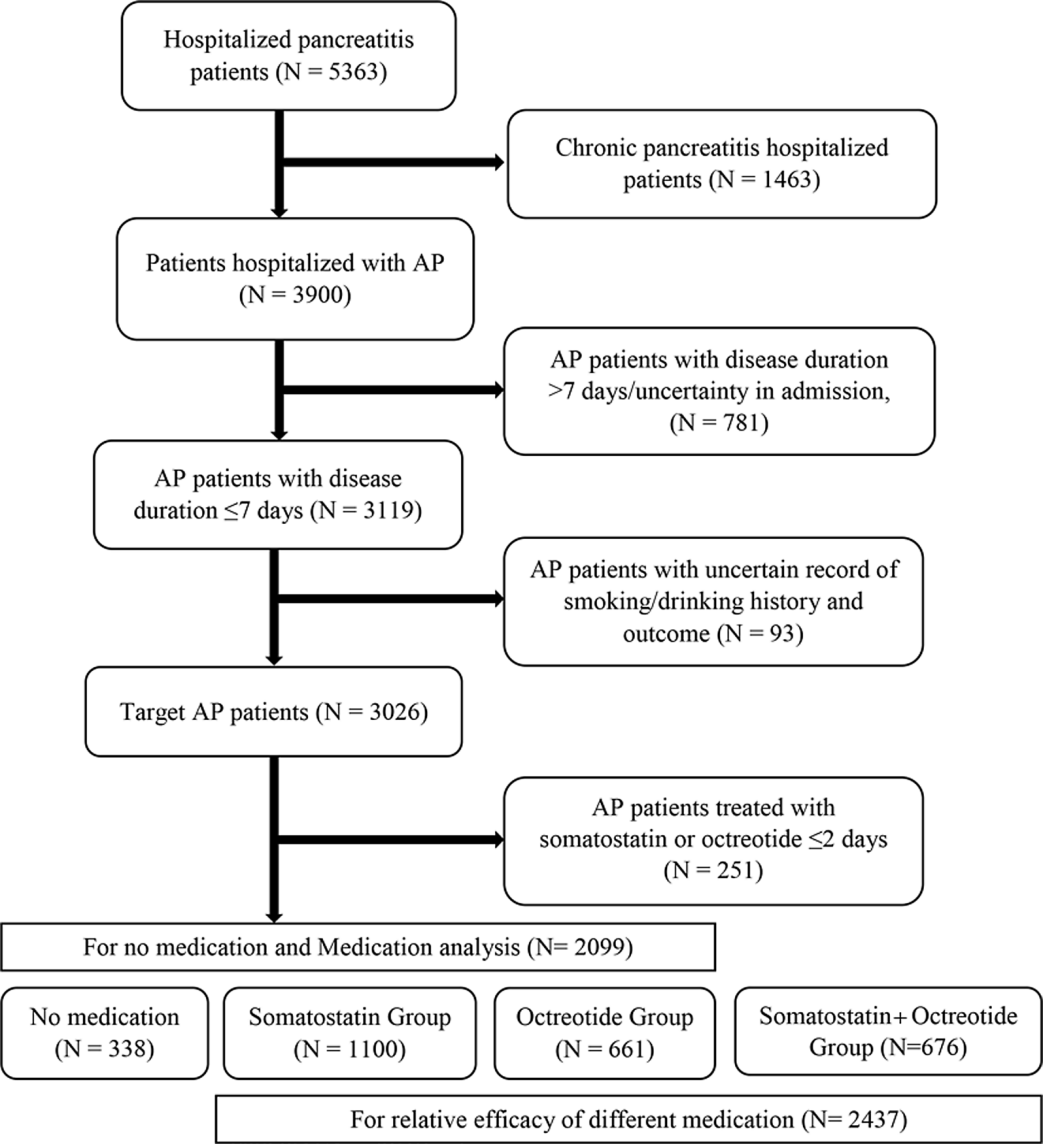
Flowchart for study population

Patients hospitalized between January 1, 2016 and December 31, 2017 with confirmative diagnosis for AP were included in this study (Fig. 1). Cases with the admission and discharge date between the pre-defined time period (January 1, 2016 to December 31, 2017) that were diagnosed of AP at admission based on ICD 10 code were included. These were used for summary of demographic characteristics. Among these, cases from patients who reported > 7 days from AP onset to diagnosis at admission to hospital or with unknown status at the time of admission, patients for whom somatostatin or octreotide was used < 3 days during hospitalization, and patients with missing key information such as drinking, smoking and clinical prognosis were excluded for further analysis on effectiveness of acid and enzyme inhibition. This study was conducted according to good clinical practices and followed all applicable regulations of the National Medical Products Administration and the principles and rules of the Ethics Committee.

### Data collection

The data extracted from the EMR database included patient demographic data, treatment & lab data, and follow-up data. Data extraction was carried out by Happy Life Science and Technology Data Department (HLT) using the Data Process & Application Platform (DPAP) account. All the data were collected from the existing clinical records of 42 departments comprising of 5,363 EMR data, 32,279 lab data (AMY/LPS) and 6,608 abdominal CT images. The details of demographic characteristics, disease characteristics, treatment, and prognosis of all eligible AP patients were extracted from the database and structured into a standard data model.

### Outcomes and endpoints

Treatment groups were divided into four based on supportive medication types for pancreatic exocrine and enzyme inhibition from medical records: somatostatin, octreotide, somatostatin + octreotide and ‘no-medication’ (no use of pancreatic exocrine and enzyme inhibitors). The effectiveness comparisons were performed at two steps: First, we compared the acid and pancreatic exocrine and enzyme inhibitors treatment groups with the no-medication group; The comparison was then conducted between the somatostatin, octreotide and somatostatin + octreotide groups. In the “somatostatin + octreotide” group, patients who received both somatostatin and octreotide during the hospital stay were included. Demographics and disease characteristics were summarized for AP status and management. Treatment outcomes were measured as incidence of disease complications and organ failure. Disease complications were defined as both local complications and systemic complications. Local complications include in detail as follow: acute fluid accumulation, acute necrotic accumulation, pancreatic pseudocyst, encapsulated necrosis, pancreatic abscess, gastrointestinal fistula; systemic complications include: organ failure, systemic inflammatory response syndrome (SIRS), systemic infection, intra-abdominal hypertension (IAH), abdominal compartment syndrome (ACS), and pancreatic encephalopathy (PE). Organ failure was defined as renal failure, respiratory failure, shock, multiple organ dysfunction syndrome (MODS) and multiple organ failure (MOF).

### Statistical analysis

Continuous data were represented as mean, median, and standard deviation. Student’s t-test and the Wilcoxon rank sum test were used for calculating statistical significance for normal and skewed distributions, respectively. Analysis of variance was used to compare statistical difference among different treatment groups for analysis of continuous data. For binary outcomes, disease complication and organ failure, the multivariate logistic regression analyses were performed to evaluate the association between drug treatment groups and each outcome. The factors associated with a prolonged length of hospital stay were determined by logarithmic linear regression analysis. The covariates in the multivariate models include drinking history, etiology, diabetes history, and severity at admission. Furthermore, propensity score matching (PSM) was performed to balance the differences in baseline characteristics as well as to reduce the effects of confounders. Logistic regression model was used to calculate the propensity scores. All statistical analyses were performed using R version 3.4.1 with a *P* < 0.05 considered as significant.

## Results

### Patient demographics before PSM analysis

During the two-year study period, a total of 5363 patients were hospitalized with pancreatitis and 3900 were diagnosed with AP. The baseline characteristics, demographic details and etiology were summarized in Table 1. Among 3900 patients, 59.7% were males and 73.2% were adults. About 25.3% of patients had known smoking and alcohol-drinking history. The most common etiology was biliary stones, which was identified in 47.7% of patients, followed by hyperlipidemia (5.8%), high-fat diet (3.1%), and alcohol induction (2.8%). The mean disease duration was 6.77 days and the median Charlson Comorbidity Index (CCI) was 2 [16]. According to severity of the disease at admission, 14.3% of patients were categorized as moderate to severe AP (MSAP and SAP) and 24.2% were categorized as MSAP and SAP at the time of discharge from the hospital based on the Chinese Acute Pancreatitis Treatment Guidelines (Draft) enacted by Study Group of Pancreatic Disease, Digestive Diseases Branch of the Chinese Medical Association in 2003.

**Table 1:** Demographic and baseline characteristics of patients

Patients before and after inclusion variable	Number of patients screened (N = 3900)
Sex: male (%)	2329 (59.7)
Age, year (mean (sd))	51.70 (17.19)
Age, years (median [IQR])	51.00 [39.00, 65.00]
Age (%)	
[0,18]	61 (1.6)
[18,65]	2854 (73.2)
[65,80]	775 (19.9)
[Above 80]	210 (5.4)
Smoking history (%)	
0	2795 (71.7)
1	987 (25.3)
Unknown	118 (3.0)
Drinking history (%)	
0	2804 (71.9)
1	988 (25.3)
Unknown	108 (2.8)
Medical history (%)	
History of pancreatitis	544 (13.9)
History of diabetes	
0	3140 (80.5)
1	501 (12.8)
Unknown	259 (6.6)
Comorbidity (%)	
With hyperlipidemia	587 (15.1)
With cholelithiasis	1229 (31.5)
Etiology (%)	
Biliary	1861 (47.7)
Hyperlipidemia	227 (5.8)
High-fat diet	120 (3.1)
Alcoholic	108 (2.8)
Duration of the disease, days (mean (sd))	6.77 (27.30)
Days of illness, days (median [IQR])	2.00 [1.00, 5.00]
Critical condition (%)	
Admitted to the hospital	559 (14.3)
Discharge of critical illness	944 (24.2)
Charlson Index (mean (sd))	2.63 (2.08)
Charlson Index (median [IQR])	2.00 [1.00, 4.00]
Standardized medication (%)	
Standard medication	3049 (78.2)
Unregulated medication	327 (8.4)
Unused	524 (13.4)
Medical treatment (%)	
Somatostatin	1600 (41.0)
Octreotide	892 (22.9)
Somatostatin octreotide	884 (22.7)
Unused	524 (13.4)
Special treatment (%)	927 (23.8)
Mechanical ventilation (%)	159 (4.1)
CRRT (%)	218 (5.6)
Local puncture drainage (%)	144 (3.7)
Laparoscopy / laparotomy (%)	356 (9.1)
ERCP (%)	268 (6.9)
Length of hospital stay, days (mean (sd))	13.97 (10.67)
Length of hospital stay, days (median [IQR])	11.00 [8.00, 17.00]
Complications at discharge (%)	351 (9.0)
Organ failure (%)	187 (4.8)
All-cause Death (%)	49 (1.3)
Enter ICU (%)	375 (9.6)

Out of 3900 patients, 781 patients with a disease course of more than 7 days or with uncertain disease course, 93 patients with missing smoking/ drinking data and 251 patients with less than 2 days of treatment, were excluded from effectiveness analysis. Among the remaining 2775 patients 2437 were divided in to three group namely somatostatin group (N = 1100), Octreotide group (N = 661) and somatostatin/Octreotide group (N = 676) and received their respective treatments and remaining 338 did not received any medication (Fig. 1). Furthermore, statistically significant difference in all baseline characteristics were observed between the groups and the details are summarized in Supplementary table 3 & 4.

### Treatment of AP

Out of 2437 patients, 1100 (45.1%) received somatostatin alone, 661 (27.1%) received octreotide, and 676 (27.7%) patients received somatostatin/octreotide (Table 1). Apart from drug therapy, 356 (9.1%) patients underwent laparoscopic/laparotomy surgery, 144 (3.7%) patients received local puncture drainage and 268 (6.9%) patients received ERCP. Further, 159 (4.1%) and 218 (5.6%) patients underwent mechanical ventilation and continuous renal replacement therapy (CRRT), respectively, for managing AP (Table 1). The median hospitalization stay was 11 (interquartile range [IQR], 8–17) days (Table 1). Also, 9.0% and 4.8% of patients experienced complications and organ failure, respectively.

### Patient demographics after PSM analysis

After PSM, a total of 1144 and 1270 propensity score matched patients were considered for somatostatin vs octreotide and somatostatin vs octreotide + somatostatin comparisons, respectively. Further, no differences in baseline clinicopathological factors were observed between the treatment groups after PSM (Supplementary table 3 & 4).

### Efficacy of pharmacologic treatment of AP

In order to assess the necessity and to compare the pharmacologic therapeutic efficacy of AP, treatment outcomes of patients who did not receive any medication (n = 338) were compared with patients who received single pharmacologic treatment (n = 1761). Patients treated with both octreotide and somatostatin were not included due to lack of data regarding the sequence of usage. While the median hospitalization days were more in patients who received medication (12 vs 9), the incidence of complications (7.6% vs 13.6%), organ failure (4.5% vs 7.4%), and the instances of entering ICU (9.3% vs 13.3%) was higher in patients in ‘no-medication’ group. Statistical significance was observed for hospitalization days, complications, organ failure and entering ICU (*P* < 0*.*05) (Supplementary Table 1).

### Complications in the different drug treatment groups

**Fig. 2:**
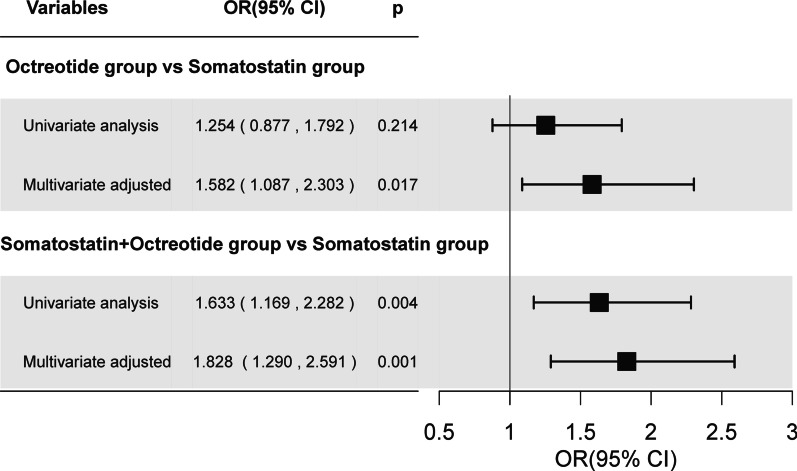
Complication risk ratio of AP patients in different treatment groups

Complications due to AP occurred in 77 (7.0%) patients in the somatostatin group, 57 (8.6%) patients in the octreotide group, and 74 (10.9%) patients in the somatostatin + octreotide group. Multivariable analysis (after controlling the confounding effects of drinking history, etiology, diabetes history, and severity at admission) revealed significant difference in the incidence of complications in the octreotide group to be 1.582 times higher than that in the somatostatin group (95% CI: 1.087–2.303, *p* = 0.017). Similarly, statistically significant difference in the incidence of complications in the somatostatin/octreotide group to be 1.828 times higher than in the somatostatin group (95% CI 1.290–2.591, *p* = 0.001) (Fig. 2). Furthermore, multivariate analysis after PSM revealed that the risk of incidence of complications in octreotide group was 2.911 times higher than in somatostatin group (95%CI 1.698, 4.989, *p* < 0.001). Similarly, a statistically significant increase in the risk of incidence of complication was observed in the somatostatin + octreotide group (OR (95% CI) 1.696 (1.125, 2.557) (*p* = 0.011) in comparison to somatostatin group (Table 2).

**Table 2:** Complication and organ failure odds ratio of AP patients in different treatment groups after propensity score matching

Variables	Complication			Organ failure		
	Event (%)	OR (95% CI)	*p*	Event (%)	OR (95% CI)	*p*
Somatostatin	19 (3.3)	1 (ref)		11 (1.9)	1 (ref)	
Octreotide	52 (9.1)	2.91 (1.69, 4.99)	< 0.001	27 (4.7)	2.53 (1.24, 5.14)	0.011
Somatostatin	40(6.3)	1 (ref)		25 (3.9)	1 (ref)	
Somatostatin + Octreotide	65(10.2)	1.70 (1.12,2.56)	0.011	38 (6.0)	1.55 (0.93,2.61)	0.093

### Organ failure in the different drug treatment groups

**Fig. 3:**
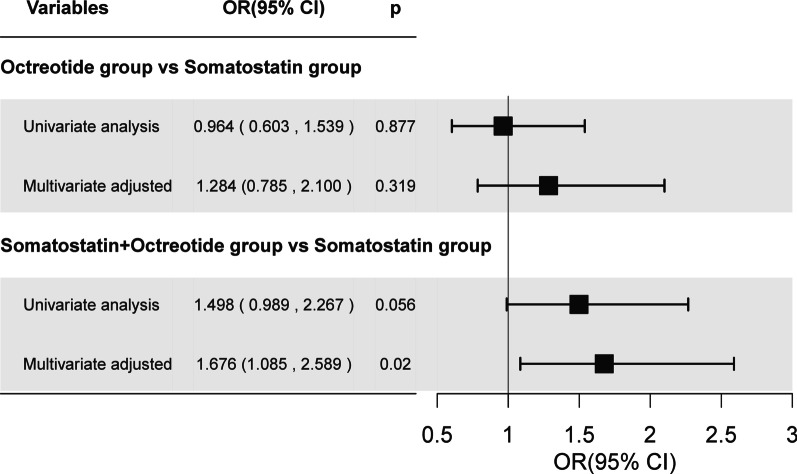
Risk ratio of organ failure in different treatment groups

Organ failure occurred in 50 (4.5%), 29 (4.4%), and 45 (6.7%) patients in the somatostatin group, the octreotide group, and the somatostatin + octreotide group, respectively. Multivariable analysis revealed the incidence of organ failure in the octreotide group to be 1.3 times higher than that in the somatostatin group (95% CI 0.785–2.100, *p* = 0.319). However, the incidence of organ failure was 1.7 times significantly higher in the somatostatin + octreotide group compared with the somatostatin group (95% CI: 1.085–2.589, *p* = 0.02) (Fig. 3). Similarly, multivariate analysis after PSM analysis revealed the incidence of organ failure in the octreotide group was significantly higher (OR 2.53, 95% CI 1.241, 5.144, *p* = 0.011)) when compared to somatostatin group. Furthermore, a non-significant increase in the incidence of organ failure was observed in somatostatin + octreotide group (OR (95% CI) 1.553 (0.926, 2.605) (*p* = 0.093) when compared with somatostatin group (Table 2).

### Duration of hospitalization stay of the different drug treatment groups

**Fig. 4:**
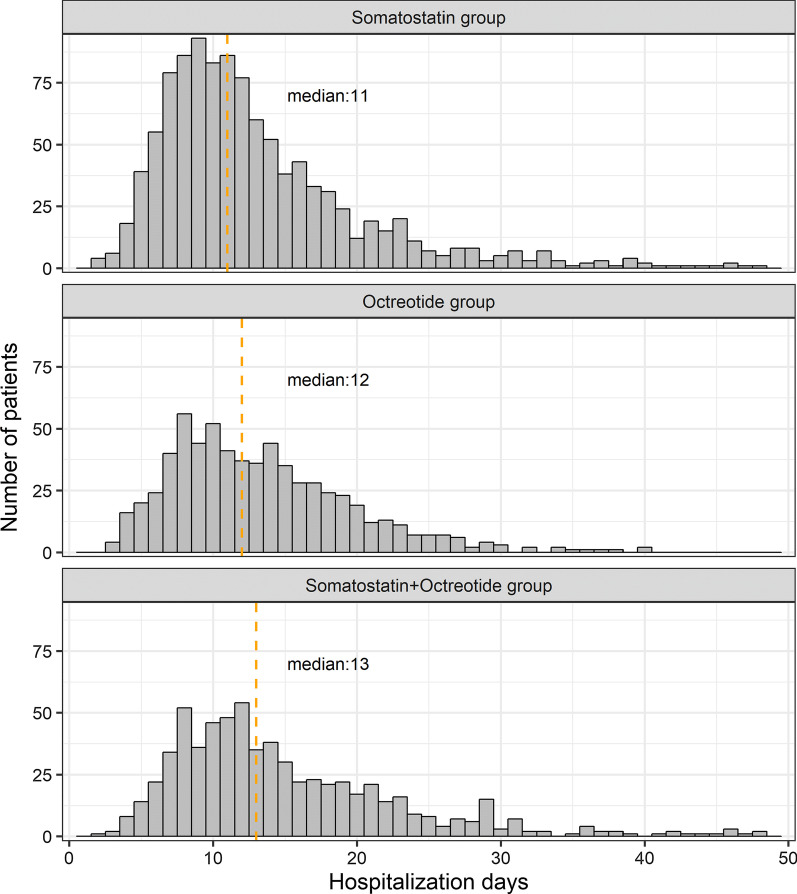
Distribution of hospitalization days in different treatment groups

Of the 2437 hospitalized AP patients, 2401 patients were included for analysis after excluding patients who died or gave up treatment during hospitalization. The median hospitalization duration was 11 days [IQR: 8–16 days] for the somatostatin group, 12 days [IQR: 9–17 days] for the octreotide group, and 13 days [IQR: 9–19 days] for the somatostatin + octreotide group. Statistically significant differences were observed among the octreotide group and the somatostatin group, the octreotide/somatostatin group and the somatostatin group (Kruskal–Wallis test *p* < 0.05) (Fig. 4). In multivariable analysis (after controlling gender, history of diabetes mellitus, duration of disease, AP severity at admission, CCI, CRRT, local puncture drainage, and laparoscopic or laparotomy), the hospitalization length of the somatostatin group was 6% and 13.9% shorter than the octreotide group (*p* = 0.015) and the somatostatin + octreotide group (*p* < 0.001), respectively (Supplementary Table 2).

### Time to amylase reaching normalcy in different treatment groups

**Fig. 5:**
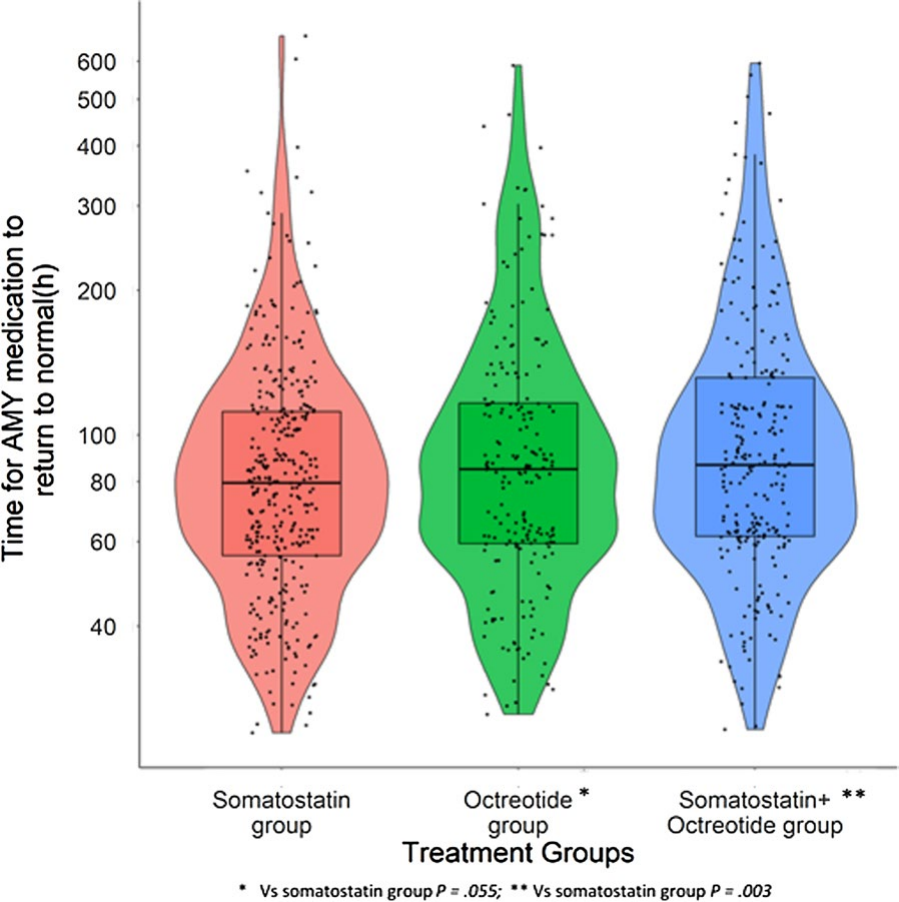
Time for AMY recovering to normal in different treatment groups

Among the 2437 AP patients, 795 patients were included for the analysis after excluding patients with normal levels of AMY or without AMY test results before initiation of treatment, and those with abnormal levels of AMY before initiation of treatment but returned to normal levels within 24 h. The recovery time of AMY was found to be 79.53 h (IQR: 56.08–113.93 h) for 351 patients in the somatostatin group, 84.88 h (IQR: 59.47–116.45 h) for 204 patients in the octreotide group, and 86.61 h (IQR: 61.54–131.73 h) for 240 patients in the somatostatin + octreotide group. In multivariable analysis (after controlling the confounding of the severity and CCI at admission), the recovery time of AMY in the somatostatin group was significantly shorter than the octreotide group (9.9%, 95% CI − 0.002–0.2%, *p* = 0.055) and the somatostatin + octreotide group (14.6%, 95% CI 0.051–0.242%, *p* = 0.003) (Fig. 5).

### Time to lipase reaching normalcy in different treatment groups

**Fig. 6:**
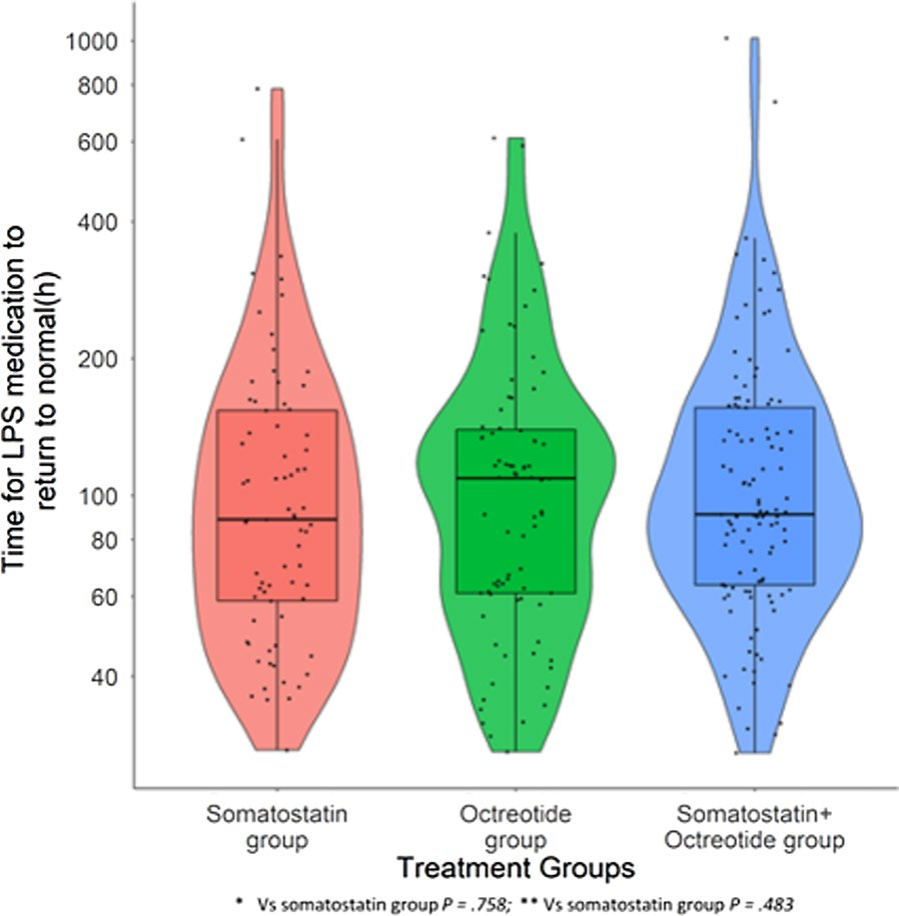
Time for LPS recovering to

A total of 265 patients were included from 2437 patients hospitalized with AP, after excluding patients with normal level of LPS or without LPS test results before initiation of treatment, or patients with abnormal levels of LPS before initiation of treatment but returned to normal levels within 24 h. LPS’s recovery time was 88.63 h (IQR: 58.68–153.63 h) for 73 patients in the somatostatin group, 109.220 h (IQR: 61.00–139.29 h) for 80 patients in the octreotide group, and 90.99 h (IQR: 63.53–155.94 h) for 112 patients in the somatostatin + octreotide group. Multivariable analysis revealed no significant difference between three treatment groups (*p* = 0.758 and *p* = 0.483 for the octreotide group and somatostatin + octreotide group compared to the somatostatin group respectively). (Fig. 6).

## Discussion

In this study, we described the current status of clinical management of AP patients and the outcomes of treatment with somatostatin and its analog, octreotide from four major geographical regions in China. We carried out detailed analysis on medical records of AP patients and the demographical, etiological parameters, risk factors and duration of hospitalization were analyzed. After PSM analysis there was no statistically significant difference in demographic characteristics among treatment groups (somatostatin vs octreotide vs somatostatin/octreotide) which increases the internal validity of the dataset. We also compared the efficacy of pharmacologic treatment of AP with non-pharmacologic management (medication vs no medication). The results of our study suggested improved outcomes in patients treated with any medication, and among the medications used somatostatin was found to provide better efficacy.

The role of pancreatic enzyme inhibitors in the treatment of AP remains unclear. However, several national clinical guidelines exist for the diagnosis and management of AP. But compliance to the recommendations is at the discretion of the treating physicians in different countries. A Japanese guidelines published in 2015, focused on the epidemiology, diagnosis, severity, treatment, post‐endoscopic retrograde cholangiopancreatography (ERCP) pancreatitis and clinical indicators but did not provide any specific recommendation on the use of pancreatic enzyme inhibitors [12]. Whereas a recent Chinese consensus statement on ERCP induced AP recommended the use of somatostatin and octreotide based on quality clinical evidence [17]. This highlights the gap in the synthesis of evidence in various consensus statement and clinical practice guidelines. The gaps in evidence base was also highlighted by the American Gastroenterological Association which stated that more studies are warranted to fulfill the knowledge gaps in the initial management of AP [14]. Hence, the results of our study intended to close the evidence gap. Irrespective of the clinical practice guideline recommendations for the management of AP [18], 45% of patients received somatostatin alone and < 10% of patients underwent special treatment such as mechanical ventilation, CRRT, ERCP, local puncture drainage, and laparoscopy in China during the study period. Owing to the multimodal approach of treating AP, therapeutic interventions other than pharmacological drugs have also been used effectively in previous studies [19]. Hence, to compare the add-on effect of somatostatin and octreotide, we compared the therapeutic outcomes in patients who were treated without medication (no medication) against patients who were treated with either somatostatin or octreotide. The analysis revealed improvement in efficacy endpoints in patients treated either with somatostatin or octreotide. While the duration of hospital stay was higher in patients undergoing treatment with somatostatin or octreotide, the incidence rate of complications, organ failure and instances of entering ICU were lower in patients receiving medication. This suggests that irrespective of other treatment modalities, pharmacologic treatment with somatostatin/octreotide should be considered for the management of AP. Contrary to our findings, a recent Cochrane review reported that pharmacologic treatment with any drug for AP did not provide consistent clinical benefits. However, the quality of evidence included were of low quality and the study included multiple treatment modalities assessing varied outcomes including short-term and long-term mortality which were not assessed in the current study [8].

In the current study, we found pancreatic exocrine and enzyme inhibition to be effective in controlling rates of complications, organ failure and instances of ICU admission. A previous study with high-dose octreotide as the intervention reported significant reduction in instances of organ failure (44% vs. 66%) [20]. Similarly, in a previous meta-analysis, among multiple drug classes, only pancreatic exocrine and enzyme inhibitors significantly reduced organ failure suggesting the superior role of somatostatin and octreotide in management of AP [8].

Investigations on somatostatin and its analogs in several experimental models of AP showed confounding results. A probable reason could be the diverse endpoints used to assess efficacy and also the underlying cause of AP in the analyzed patients. Since the clinical decision of managing AP in real-world settings is made on a case-to-case basis, there are practical issues with conducting randomized controlled trials evaluating somatostatin and octreotide [21]. The efficacy of somatostatin in AP patients has been proven in 7-day multicenter randomized trial in 1980 [22]. Still there is a discrepancy persisting on the administration of standard medication that is associated with the reduction of mortality and morbidity of AP patients [11]. Although we found somatostatin to be effective than octreotide, the probable reason for the observed trend could not be ascertained in the current study.

With recent advancement in treatment [6], the length of hospitalization stay significantly decreased across all the time points in the earlier decades [7]. Our study also demonstrated similar reduction in hospitalization days. In agreement with the previous trials reviewed by Li et al. the incidence of complications associated with AP was found to be lesser in the somatostatin group compared with the octreotide group and the somatostatin + octreotide group [9], The proportion of organ failure was low in the octreotide group, which is in accordance with previous trials conducted for the octreotide group, than in control [23]. As somatostatin analogs are pancreatic enzyme inhibitors [24], the recovery time for serum AMY/LPS was shorter in somatostatin-treated patients than that in the octreotide-treated patients, suggesting rapid recovery of AP patients treated with somatostatin. Investigations on somatostatin and its analogs in several experimental models of AP showed confounding results. A probable reason could be the diverse endpoints used to assess efficacy and also the underlying cause of AP in the analyzed patients. Since the clinical decision of managing AP in real-world settings is made on a case-to-case basis, there are practical issues with conducting randomized controlled trials evaluating somatostatin and octreotide [21]. The efficacy of somatostatin in AP patients has been proven in 7-day multicenter randomized trial in 1980 [22]. Still there is a discrepancy persisting on the administration of standard medication that is associated with the reduction of mortality and morbidity of AP patients [11].

## Conclusion

To the best of our knowledge, this is the first study to use extensive data generated by big data analysis platform’s uniform EMR for the management of AP in China. This real-world data provided high-quality evidence in the current status of management of AP patients in China. Further, there are no real-world data available for the comparison of somatostatin, octreotide, and somatostatin + octreotide in the pharmacologic management of AP. Based on this real-world evidence from China, somatostatin might be effective than octreotide with respect to both the efficacy and complications at discharge in the patients hospitalized with AP.

### Strength and limitations

The main strength of the study is large and real-world data from different regions of China used for the analysis. This broader the view of risk–benefit profile of somatostatin analogs used in the management of AP. The endpoints analyzed were comprehensive encompassing both efficacy and current status of patients with AP in China.

Some limitations should be noted when interpreting the results. First, some patients with key information missing were excluded from the analysis, which led to a reduction in the sample size. Another limitation relates to the retrospective nature of the study and limited information recorded in the system; for example, dosage details of medication and meal start time were not captured by the electronic health record in a comprehensive manner, which prevented further assessment and might have an impact on the results to some degree.

## Supplementary Information

**Additional file 1.** **Supplementary Table 1.** Therapeutic Outcomes in Patients Treated With and Without acid and enzyme inhibition Medication. **Supplementary Table 2.** Hospitalization Days in Different Treatment Groups (Multiple Linear Regression Analysis). **Supplementary Table 3.** Matched baseline characteristics comparison between somatostatin vs octreotide in propensity score matching analysis. **Supplementary Table 4.** Matched baseline characteristics comparison between somatostatin vs octreotide+somatostatin in propensity score matching analysis.

## Data Availability

The datasets generated and/or analyzed during the current study are not publicly available due to data confidentiality but are available from the corresponding author on reasonable request.
