# Supplementary material for: Current diagnosis and treatment of acute pancreatitis in China: a real-world, multicenter study
Source: BMC Gastroenterol. 2021 May 8;21:210. doi: 10.1186/s12876-021-01799-1 (PMC8105912; doi:10.1186/s12876-021-01799-1)
Supplement: Supplementary file 1 — Additional file 1. Supplementary Table 1. Therapeutic Outcomes in Patients Treated With and Without acid and enzyme inhibition Medication. Supplementary Table 2. Hospitalization Days in Different Treatment Groups (Multiple Linear Regression Analysis). Supplementary Table 3. Matched baseline characteristics comparison between somatostatin vs octreotide in propensity score matching analysis. Supplementary Table 4. Matched baseline characteristics comparison between somatostatin vs octreotide+somatostatin in propensity score matching analysis. [file 12876_2021_1799_MOESM1_ESM.docx]

**Supplementary Table 1.** Therapeutic Outcomes in Patients Treated with and Without acid and enzyme inhibition Medication

| **Variable** | **Standardized Medication**  **(N=1761)** | **No Medication**  **(N=338)** | ***P*** |
| --- | --- | --- | --- |
|  |  |  |  |
| Length of hospital stay, days (mean (sd)) | 13.83 (9.65) | 11.67 (8.38) | <.001 |
| Length of hospital stay, days (median [IQR]) | 12 [8, 17] | 9 [6, 14] | <.001 |
| Complications at discharge (%) | 134 (7.6) | 46 (13.6) | <.001 |
| Organ failure (%) | 79 (4.5) | 25 (7.4) | .024 |
| All-cause death (%) | 31 (1.8) | 1 (0.3) | .044 |
| Enter ICU (%) | 164 (9.3) | 45 (13.3) | .024 |

**Supplementary Table 2.** Hospitalization Days in Different Treatment Groups (Multiple Linear Regression Analysis)

| **Variables** | **β** | **95% CI** | ***P*** |  |
| --- | --- | --- | --- | --- |
| Medication groups |  |  |  |  |
| Somatostatin group | *Ref* | *Ref* | *Ref* | |
| Octreotide group | 0.06 | (0.012, 0.108) | 0.015 | |
| Somatostatin + Octreotide group | 0.135 | (0.087, 0.182) | <0.001 | |
| Other Factors |  |  |  |  |
| Gender, male | -0.101 | ( -0.141, -0.061) | <0.001 | |
| Diabetes | 0.097 | (0.055, 0.138) | <0.001 | |
| Duration of disease, days | 0.012 | (0.002, 0.023) | 0.021 | |
| MSAP, admission | 0.296 | (0.239, 0.353) | <0.001 | |
| CCI | 0.013 | (0.003, 0.022) | 0.012 | |
| CRRT | 0.298 | (0.207, 0.388) | <0.001 | |
| Local puncture drainage | 0.476 | (0.361, 0.591) | <0.001 | |
| Laparotomy | 0.415 | (0.347, 0.483) | <0.001 | |

**Supplementary Table 3.** Matched baseline characteristics comparison between somatostatin vs octreotide in propensity score matching analysis

|  | **Pre-matching** | | | **Post-matching** | | |
| --- | --- | --- | --- | --- | --- | --- |
| **Variables** | **Somatostatin**  **（N=1100）** | **Octreotide**  **（N=661）** | ***P*** | **Somatostatin**  **（N=572）** | **Octreotide**  **（N=572）** | ***P*** |
| **Sex: Male (%)** | 637(57.9) | 396(59.9) | 0.409 | 333 (58.2) | 330 (57.7) | 0.857 |
| **Age, year (mean(std))** | 50.55(16.66) | 51.99(15.86) | 0.075 | 50.97 (16.11) | 51.52 (16.03) | 0.564 |
| **Age (%)** |  |  |  |  |  |  |
| **(0,18)** | 9(0.8) | 3(0.5) | 0.094 | 4 (0.7) | 3 (0.5) | 0.723 |
| **(18,65)** | 839(76.3) | 493(74.6) |  | 438 (76.6) | 432 (75.5) |  |
| **(65,80)** | 192(17.5) | 140(21.2) |  | 112 (19.6) | 112 (19.6) |  |
| **(80 Above)** | 60(5.5) | 25(3.8) |  | 18 (3.1) | 25 (4.4) |  |
| **Smoking history (%)** | 294(26.7) | 147(22.2) | 0.035 | 135 (23.6) | 133 (23.3) | 0.889 |
| **alcohol history (%)** | 304(27.6) | 137(20.7) | 0.001 | 138 (24.1) | 126 (22.0) | 0.4 |
| **History of pancreatitis (%)** | 175(15.9) | 60(9.1) | <0.001 | 67 (11.7) | 55 (9.6) | 0.25 |
| **History of diabetes (%)** | 152(13.8) | 78(11.8) | 0.225 | 75 (13.1) | 74 (12.9) | 0.964 |
| **Comorbidity (%)** |  |  |  |  |  |  |
| **With hyperlipidemia (%)** | 164(14.9) | 128(19.4) | 0.015 | 104 (18.2) | 93 (16.3) | 0.389 |
| **With cholelithiasis (%)** | 345(31.4) | 220(33.3) | 0.403 | 191 (33.4) | 191 (33.4) | 1 |
| **Etiology (%)** |  |  |  |  |  |  |
| **Biliary (%)** | 486(44.2) | 335(50.7) | 0.008 | 289 (50.5) | 282 (49.3) | 0.679 |
| **Hyperlipidemia (%)** | 109(9.9) | 22(3.3) | <0.001 | 23 (4.0) | 22 (3.8) | 0.879 |
| **High fat diet (%)** | 44(4.0) | 19(2.9) | 0.218 | 15 (2.6) | 19 (3.3) | 0.486 |
| **Alcoholic (%)** | 24(2.2) | 16(2.4) | 0.745 | 16 (2.8) | 14 (2.4) | 0.711 |
| **Diabetes (%)** | 803(73.0) | 329(49.8) | <0.001 | 329 (57.5) | 315 (55.1) | 0.404 |
| **Days of illness, days (median [IQR])** | 1[1,3] | 2[1,3] | <0.001 | 1[1,3] | 2[1,3] | 0.082 |
| **Admission with critical condition (%)** | 204(18.5) | 76(11.5) | <0.001 | 74 (12.9) | 71 (12.4) | 0.79 |
| **Charlson Index (median [IQR])** | 2[1,4] | 2[1,4] | 0.002 | 2[1,4] | 2[1,4] | 0.597 |
| **Mechanical Ventilation (%)** | 57(5.2) | 16(2.4) | 0.005 | 17 (3.0) | 16 (2.8) | 0.86 |
| **CRRT (%)** | 82(7.5) | 17(2.6) | <0.001 | 14 (2.4) | 17 (3.0) | 0.585 |
| **Local puncture drainage (%)** | 19(1.7) | 20(3.0) | 0.073 | 12 (2.1) | 14 (2.4) | 0.692 |
| **Laparoscopy / Laparotomy (%)** | 93(8.5) | 68(10.3) | 0.196 | 58 (10.1) | 60 (10.5) | 0.846 |

**Supplementary Table 4.** Matched baseline characteristics comparison between somatostatin vs octreotide+somatostatin in propensity score matching analysis

|  | **Pre-matching** | | | **Post-matching** | | |
| --- | --- | --- | --- | --- | --- | --- |
| **Variables** | **Somatostatin** | **Octreotide**  **+**  **Somatostatin** | ***P*** | **Somatostatin** | **Octreotide**  **+**  **Somatostatin** | ***P*** |
|  | **(N=1100）** | **(N=676）** |  | **(N=635）** | **(N=635）** |  |
| **Sex: Male (%)** | 637(57.9) | 403(59.6) | 0.478 | 375(59.1) | 375(59.1) | 1 |
| **Age, year (mean(std))** | 50.55(16.66) | 52.98(17.01) | 0.003 | 52.44(17.12) | 52.67(17.00) | 0.808 |
| **Age (%)** |  |  | 0.22 |  |  | 0.879 |
| **[0,18)** | 9(0.8) | 3(0.4) |  | 5(0.8) | 3(0.5) |  |
| **[18,65)** | 839(76.3) | 491(72.6) |  | 455(71.7) | 463(72.9) |  |
| **[65,80)** | 192(17.5) | 137(20.3) |  | 134(21.1) | 129(20.3) |  |
| **[80 above)** | 60(5.5) | 45(6.7) |  | 41(6.5) | 40(6.3) |  |
| **Smoking history (%)** | 294(26.7) | 164(24.3) | 0.249 | 165(26.0) | 160(25.2) | 0.748 |
| **Drinking history (%)** | 304(27.6) | 168(24.9) | 0.197 | 164(25.8) | 159(25.0) | 0.747 |
| **History of pancreatitis (%)** | 175(15.9) | 74(10.9) | 0.003 | 79(12.4) | 74(11.7) | 0.666 |
| **History of diabetes (%)** | 152(13.8) | 93(13.8) | 0.627 | 82(12.9) | 90(14.2) | 0.793 |
| **Comorbidity (%)** |  |  |  |  |  |  |
| **With hyperlipidemia (%)** | 164(14.9) | 136(20.1) | 0.004 | 117(18.4) | 120(18.9) | 0.829 |
| **With cholelithiasis (%)** | 345(31.4) | 241(35.7) | 0.062 | 233(36.7) | 230(36.2) | 0.861 |
| **Etiology (%)** |  |  |  |  |  |  |
| **Biliary (%)** | 486(44.2) | 374(55.3) | <0.001 | 341(53.7) | 343(54.0) | 0.91 |
| **Hyperlipidemia (%)** | 109(9.9) | 27(4.0) | <0.001 | 28(4.4) | 27(4.3) | 0.89 |
| **High fat die t(%)** | 44(4.0) | 29(4.3) | 0.765 | 27(4.3) | 27(4.3) | 1 |
| **Alcoholic (%)** | 24(2.2) | 27(4.0) | 0.026 | 21(3.3) | 20(3.1) | 0.874 |
| **Diabetes (%)** | 803(73.0) | 425(62.9) | <0.001 | 424(66.8) | 410(64.6) | 0.408 |
| **Days of illness, days (median [IQR])** | 1[1,3] | 2[1,4] | <0.001 | 2[1,3] | 2[1,3] | 0.139 |
| **Admission with critical condition (%)** | 204(18.5) | 101(14.9) | 0.051 | 97(15.3) | 95(15.0) | 0.876 |
| **Charlson Index (median [IQR])** | 2[1,4] | 3[1,4] | <0.001 | 3[1,4] | 3[1,4] | 0.694 |
| **Mechanical Ventilation (%)** | 57(5.2) | 36(5.3) | 0.895 | 27(4.3) | 30(4.7) | 0.684 |
| **CRRT (%)** | 82(7.5) | 41(6.1) | 0.263 | 42(6.6) | 37(5.8) | 0.561 |
| **Local puncture drainage (%)** | 19(1.7) | 36(5.3) | <0.001 | 19(3.0) | 19(3.0) | 1 |
| **Laparoscopy / Laparotomy (%)** | 93(8.5) | 60(8.9) | 0.759 | 56(8.8) | 59(9.3) | 0.769 |
